# Interferon Gamma Induces Changes in Natural Killer (NK) Cell Ligand Expression and Alters NK Cell-Mediated Lysis of Pediatric Cancer Cell Lines

**DOI:** 10.3389/fimmu.2017.00391

**Published:** 2017-04-06

**Authors:** Arianexys Aquino-López, Vladimir V. Senyukov, Zlatko Vlasic, Eugenie S. Kleinerman, Dean A. Lee

**Affiliations:** ^1^Department of Pediatric Research, The University of Texas M.D. Anderson Cancer Center, Graduate School of Biomedical Sciences, Houston, TX, USA; ^2^School of Medicine, University of Puerto Rico, San Juan, Puerto Rico; ^3^Center for Childhood Cancer and Blood Disorders, The Research Institute, Nationwide Children’s Hospital, Columbus, OH, USA

**Keywords:** interferon gamma, natural killer cells, pediatric cancer, mass cytometry, natural killer ligands, immunotherapy, intercellular adhesion molecule 1, major histocompatibility complex

## Abstract

Natural killer (NK) cells have therapeutic potential for cancer due to their capacity for targeting tumor cells without prior sensitization. Our laboratory has developed an NK cell expansion protocol that generates large quantities of NK cells for therapeutic infusion that secret 20 times the amount of interferon gamma (IFNγ) than resting NK cells. IFNγ can upregulate major histocompatibility complex (MHC)-class I, an inhibitory ligand for NK cells, but can also upregulate intercellular adhesion molecule 1 (ICAM-1) which promotes NK:target cell interaction for an efficient lysis. Due to the opposing effects reported for IFNγ on tumor sensitivity to NK cells, we evaluated a panel 22 tumor cell lines from the pediatric preclinical testing program corresponding to different tumor types. We determined the impact of IFNγ on their expression of NK cell activating and inhibitory ligands, death receptors, and adhesion molecules using mass cytometry. We also evaluated the effect of IFNγ on their sensitivity to NK cell-mediated lysis. Our results show upregulation of PD-L1, ICAM-1, MHC-class I, HLA-DR, CD95/FasR, and CD270/HVEM after IFNγ treatment, this upregulation is variable across different tumor types. We also observed a variable impact of IFNγ in NK cell-mediated lysis. For six of the cancer cell lines IFNγ resulted in increased resistance to NK cells, while for three of them it resulted in increased sensitivity. Modeling of the data suggests that the effect of IFNγ on NK cell-mediated tumor lysis is mostly dependent on changes in MHC-class I and ICAM-1 expression. For three of the cell lines with increased resistance, we observed higher upregulation of MHC-class I than ICAM-1. For the cell lines with increased sensitivity after IFNγ treatment, we observed upregulation of ICAM-1 exceeding MHC-class I upregulation. ICAM-1 upregulation resulted in increased conjugate formation between the NK cells and tumor cells, which can contribute to the increased sensitivity observed. However, the effects of MHC-class I and ICAM-1 are not readily predictable. Due to the high IFNγ secretion of NK cell infusion products, a better understanding of the NK ligands on tumor cells and how they are affected by IFNγ is essential to optimize NK cell immunotherapy.

## Introduction

Current chemotherapeutic approaches for pediatric cancer are associated with high morbidity and late effects in survivors. By the age of 50, many survivors experience cardiovascular, renal, and hepatic complications, in addition to the risk of subsequent malignancies (1). Therefore, there is a need to develop new therapies capable of eradicating disease with reduced late effects. Immunotherapy has emerged as a promising new approach for cancer treatment. Natural killer (NK) cells, an important component of our first-line innate immunity, have activity against tumor cells without prior sensitization and are increasingly recognized for their important role in preventing and eradicating cancer. Because NK cells have the ability to selectively target tumor cells without affecting healthy cells, they are an attractive approach for pediatric cancer therapy.

Natural killer cells are typically defined as CD56+/CD3− lymphocytes. Their activity is regulated by a series of inhibitory and activating receptors that recognize ligands on target cells. The balance between activating and inhibitory signals will determine whether an NK cell is activated. The three main receptor families present on NK cells include natural cytotoxicity receptors (NCRs), C-type lectin (CD94/NKG2), and killer cell immunoglobulin-like receptors (KIRs) (2). NCRs (NKp30, NKp44, NKp46) are activating receptors, as are CD244/2B4, CD226/DNAM-1, and CD314/NKG2D. KIRs and C-type lectin receptors may be activating or inhibitory. Among the most important NK cell, inhibitory receptors are the KIRs and NKG2A because they recognize major histocompatibility complex (MHC) on target cells as evidence of self, leading to inhibition of NK cell activity (3). Many tumor cells downregulate MHC to escape T cell immunity, and upregulate activating ligands, making them susceptible targets for NK cell attack (4–7). However, in tumor cells where both inhibitory and activating ligands are present, the balance of these signals determines whether the NK cell is activated.

We developed an expansion protocol that allows production of large quantities of NK cells for adoptive immunotherapy (8). We have observed that, as compared to primary NK cells or IL-15-expanded NK cells, IL-21-expanded NK cells secrete 20-fold or 100-fold more interferon gamma (IFNγ, median 2,493 vs. 24 or 111 pg/mL, respectively), in response to target recognition (8). IFNγ has been reported to upregulate the inhibitory MHC-class I in target cells, making them more resistant to NK cell-mediated lysis (9, 10). However, IFNγ has also been reported to promote NK:target cell interaction through upregulation of intercellular adhesion molecule 1 (ICAM-1), promoting increased target cell death (11, 12). These findings suggest that IFNγ can have opposing effects on tumor cell sensitivity to NK cell-mediated lysis. To optimize the use of expanded NK cells as an immunotherapy it is imperative that we better understand how IFNγ affects NK cell-mediated lysis of the tumor cells.

Opposing effects reported for IFNγ may be due to a focus on specific tumor types, therefore this study aimed to evaluate the effect of IFNγ on a broad selection of 22 tumor cell lines from the pediatric preclinical testing program (PPTP) *in vitro* panel. This panel was designed to evaluate new therapies against childhood leukemias and solid tumors and has already been used for *in vitro* testing of over 50 pediatric cancer therapies (13). Using these cancer cells corresponding to six different types of pediatric malignancies, we evaluated the effects of IFNγ treatment in tumor cell sensitivity to NK cell-mediated lysis. Also we evaluated the effects of IFNγ treatment on tumor expression of NK cell ligands, including activating and inhibitory ligands, death receptors, and adhesion molecules.

## Materials and Methods

### Isolation and Expansion of Human NK Cells

Buffy coats from four anonymized donors were obtained from Gulf Coast Regional Blood Center (Houston, TX, USA). Exemption and waiver of consent for the research use of buffy coat fractions obtained from anonymized donors at Gulf Coast Regional Blood Center (Houston, TX, USA) was granted by the Institutional Review Board of the University of Texas MD Anderson Cancer Center under protocol PA13-0978. NK cells were isolated using the RossetteSep Human NK cell enrichment cocktail (Stem Cell Technologies) and expanded as described previously using K562 Clone9.mbIL21 as feeder cells for 21 days (8). Expanded NK cells were cryopreserved, and subsequently thawed and recovered for 1–2 days prior to their use. During recovery NK cells were cultured in NK cell media consisting of RPMI 1640 (Corning) supplemented with 50 IU/mL recombinant human IL-2 (Proleukin, Novartis Vaccines and Diagnostics, Inc.), 20% Fetal Bovine Serum (Thermofisher), l-glutamine (Gibco), and penicillin/streptomycin (Corning).

### Tumor Cells

TC-71, NALM-6, and Ramos-RA1 were obtained as kind gifts from colleagues (Drs. Eugenie S. Kleinerman, L. J. N. Cooper, and J. Chandra, respectively). Karpas-299 was obtained from the German Collection of Microorganisms and Cell Cultures (DSMZ). RS4;11, MOLT-4, and CCRF-CEM were obtained from the America Type Culture Collection (ATCC). The remaining cell lines were obtained from the Children’s Oncology Group (COG) Cell Line and Xenograft Repository. Brain tumor cell lines BT-12, SJ-GBM2, CHLA-266, Ewing sarcoma (EWS) cell lines CHLA-9, CHLA-10, CHLA-258, TC-71, neuroblastoma (NB) cell lines NB1643, NB-EBc1, CHLA-90, CHLA-136, rhabdomyosarcoma (RMS) cell line RD, and leukemia cell line COG-LL-317 were cultured in IMDM (Lonza) supplemented with 20% FBS (Thermofisher), 4 mM l-glutamine (Gibco), 1× ITS (Lonza), and penicillin/streptomycin (Corning). Lymphoma cell lines Karpas-299, Ramos-RA1, leukemia cell lines NALM-6, RS4;11, MOLT-4, CCRF-CEM, Kasumi-1, and RMS cell lines Rh41, Rh30, were cultured in RPMI 1640 (Corning) supplemented with 10% Fetal Bovine Serum (Thermofisher), l-glutamine (Gibco), and penicillin/streptomycin (Corning). Cultures were periodically tested to confirm absence of mycoplasma using MycoAlert Mycoplasma Detection Kit (Lonza). Identity was confirmed by STR DNA fingerprinting either using the AmpFlSTR Identifiler kit (Applied Biosystems) or the Power Plex 16HS Kit (Promega) according to manufacturer instructions. The STR profiles were compared to known fingerprints as published by ATCC or the COG cell STR Genotype Database (http://strdb.cogcell.org). STR profiles were last performed on March 2016 (SJ-GBM2, NB1643, MOLT-4), October 2015 (RD, Rh41, Rh30, BT-12, CHLA-10, NB-EBc1, NALM-6, and Ramos-RA1), or September 2012 (CHLA-266, CHLA-9, CHLA-258, TC-71, CHLA-90, CHLA-136, RS4;11, COG-LL-317, CCRF-CEM, Kasumi-1, and Karpas-299). Banks of STR validated, mycoplasma-free cell lines were cryopreserved. Cell lines were kept in culture no longer than eight passages or 4 weeks prior to use.

### IFNγ Treatment of Tumor Cells

Cell lines that grow in suspension were seeded at 0.5 × 10^6^ cells/mL and treated with 50 ng/mL of IFNγ (Peprotech) for 48 h. Adherent cells were cultured to a 60–70% confluence and treated with 50 ng/mL of IFNγ (Peprotech) for 48 h. Untreated tumor cells were seeded in parallel. After treatment cells were washed in IFNγ free media, and adherent cells were detached with non-enzymatic cell dissociation buffer (Gibco) to avoid degradation of cell surface proteins. Treated and untreated cells were evaluated for surface expression of NK cell ligands by mass cytometry and sensitivity to NK cell-mediated lysis by calcein release assay.

### Cytotoxicity

The fluorescence based calcein release assay was used to assess cytotoxicity, as previously described (8, 14). Adherent cells were detached with non-enzymatic cell dissociation buffer (Gibco) and cells were filtered by using a 70 μm cell strainer (Corning) to obtain a single-cell suspension. Target cells were labeled with 5 μg/mL of calcein-AM (Sigma-Aldrich) for 1 h at 37°C. NK cells were cocultured with target cells at different effector to target (E:T) ratios (10:1, 5:1, 2.5:1, 1.25:1, 0.6:1, and 0.3:1) for 4 h at 37°C. Supernatant fluorescence was determined at 485 nm^Exc^/530 nm^Emm^ using the SpectraMax Plus^384^ spectrophotometer.

### Mass Cytometry

Antibodies for mass cytometry were labeled with heavy metals using Maxpar-X8 labeling reagent kits (DVS Sciences) according to manufacturer’s instructions and titrated for determination of optimal concentration. The antibodies and their respective heavy metal labeling can be found in Table S1 in Supplementary Material. Since NK cell receptors may have multiple ligands (e.g., NKG2D binds to MICA, MICB, and ULBP1-5), or unknown ligands, chimeric receptor:IgG-Fc fusion proteins were tagged with heavy metals and used for identification of ligands on tumor cells. Then, 1.5 × 10^6^ cells were stained for viability with 2.5 µM cell ID cisplatin (Fluidigm, 201064) in serum free RPMI for 1 min and washed twice with complete media. Subsequently, surface staining was performed as previously described (15). Staining media were prepared by adding 5% FBS and 0.1% sodium-azide to PBS. During the intracellular staining step of tumor cells, two different isotopes of cisplatin Pt-194 (Fluidigm, 201194) and Pt-198 (Fluidigm, 201198) were used to barcode untreated and IFNγ-treated samples, respectively, allowing samples to be combined in a single tube, minimizing acquisition time, and variability between runs. Data were acquired on a CyTOF instrument (DVS Sciences). Files containing only live single cells were exported using FlowJo V10 Software and uploaded into Cytobank for further analysis (Figure S1 in Supplementary Material) (16).

### Conjugation Assay

The determination of effector conjugation to target cells was performed as described by Burshtyn et al., with some minor modifications (17). Briefly, NK cells and tumor cells were stained with green dye PKH67-GL (Sigma, MINI67) and red dye PKH26-GL (Sigma, MINI26), respectively, in 5 µM dye at 5 × 10^6^ cells/mL for 5 min at room temperature. Dye staining was stopped by adding two volumes of FBS and two volumes of complete media. Cells were washed twice with complete media and let rest for at least 1 h at 37°C. Then, 10^5^ NK cells were combined with 2 × 10^5^ tumor cells in 200 µL, centrifuged at 20 *g* for 1 min (to initiate contact), and incubated at 37°C for 30 min. Cells were resuspended by gentle vortexing, fixed with 200 µL of 4% formaldehyde, and analyzed by flow cytometry. For antibody blocking experiments, NK cells were pre-incubated with 5 µL of Fc blocker (Biolegend, 422302) for 10 min to avoid antibody-dependent cell cytotoxicity. ICAM-1 was blocked on tumor cells by adding 10 µg/mL of anti-CD54/ICAM-1 clone HCD54 (Biolegend, 322703) for 20 min at room temperature.

### Statistical Analysis

**Figure 1:**
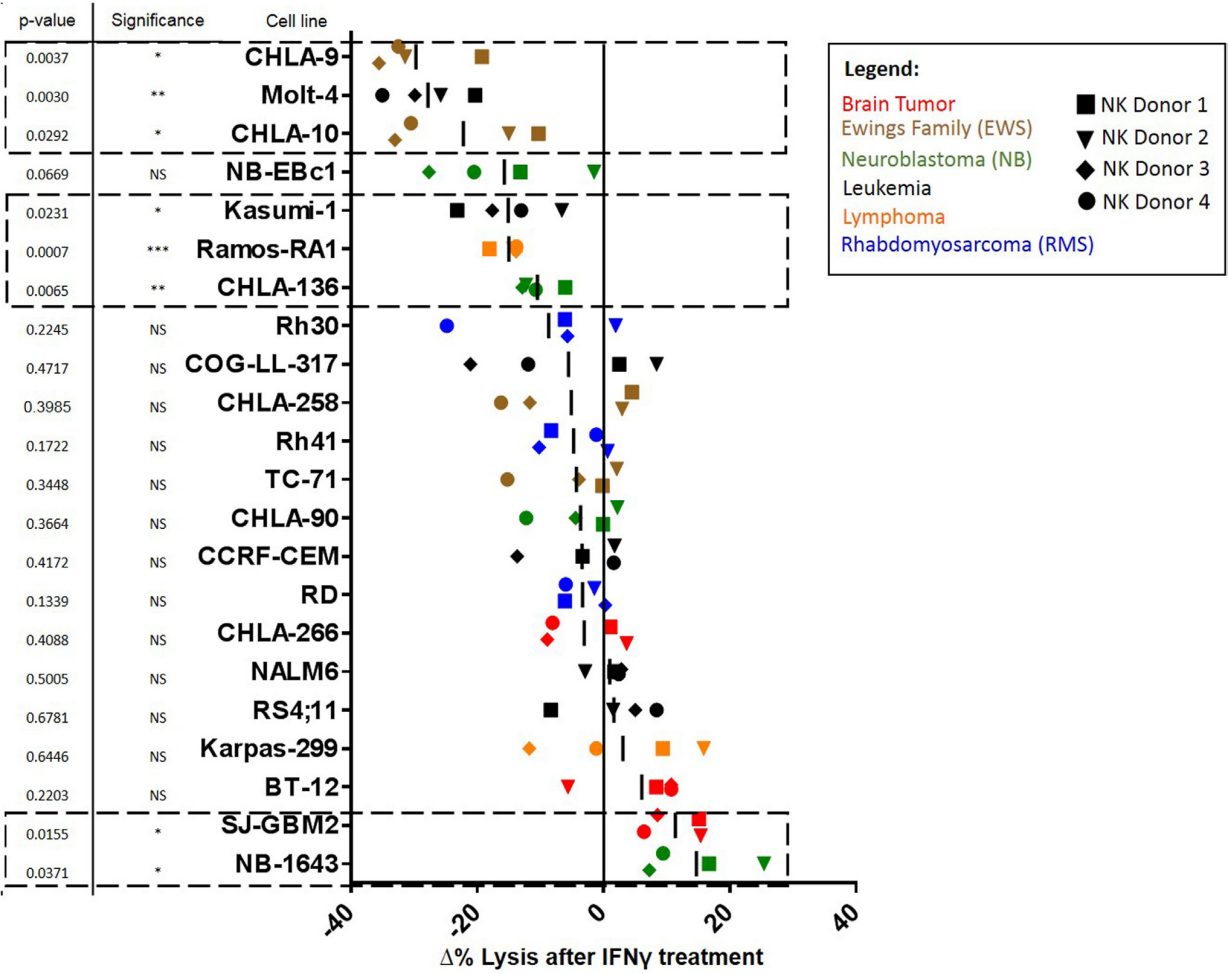
**Waterfall plot of change in lysis by natural killer (NK) cells after treatment of pediatric cancer cell lines with IFNγ**. NK cell cytotoxic activity toward IFNγ-treated and -untreated cancer cells was evaluated using calcein release assays. Changes in lysis (Δ% Lysis) of treated compared to untreated cancer cells were quantified for each NK cell donor. Each shape represents a different NK cell donor (the same four donors were used for all cell lines). Color coding corresponds to cell line cancer type. *p*-Values are for probability that Δ% ≠ 0 (*t*-test). Squares with dashed lines indicate cancer cell lines with significant (*p* ≤ 0.05) changes in NK cell-mediated lysis after IFNγ treatment.

Statistics were performed in GraphPad Prism Software. For determination of Δ in % Lysis after IFNγ treatment (Figure 1), we used data from six different E:T ratios. For each cell line, we calculated the average difference in lysis after IFNγ treatment (%lysis IFNγ treated − %lysis untreated) using four NK cell donors. Significance was determined by using the *t*-test with a hypothetical value of 0 for comparison. Heatmaps were generated using Cytobank (16). Conjugation data significance was determined by using the *t*-test, statistical significance was determined by a *p* < 0.05.

## Results

### IFNγ Has a Variable Impact on Tumor Cell Sensitivity to NK Cell-Mediated Lysis

**Figure 2:**
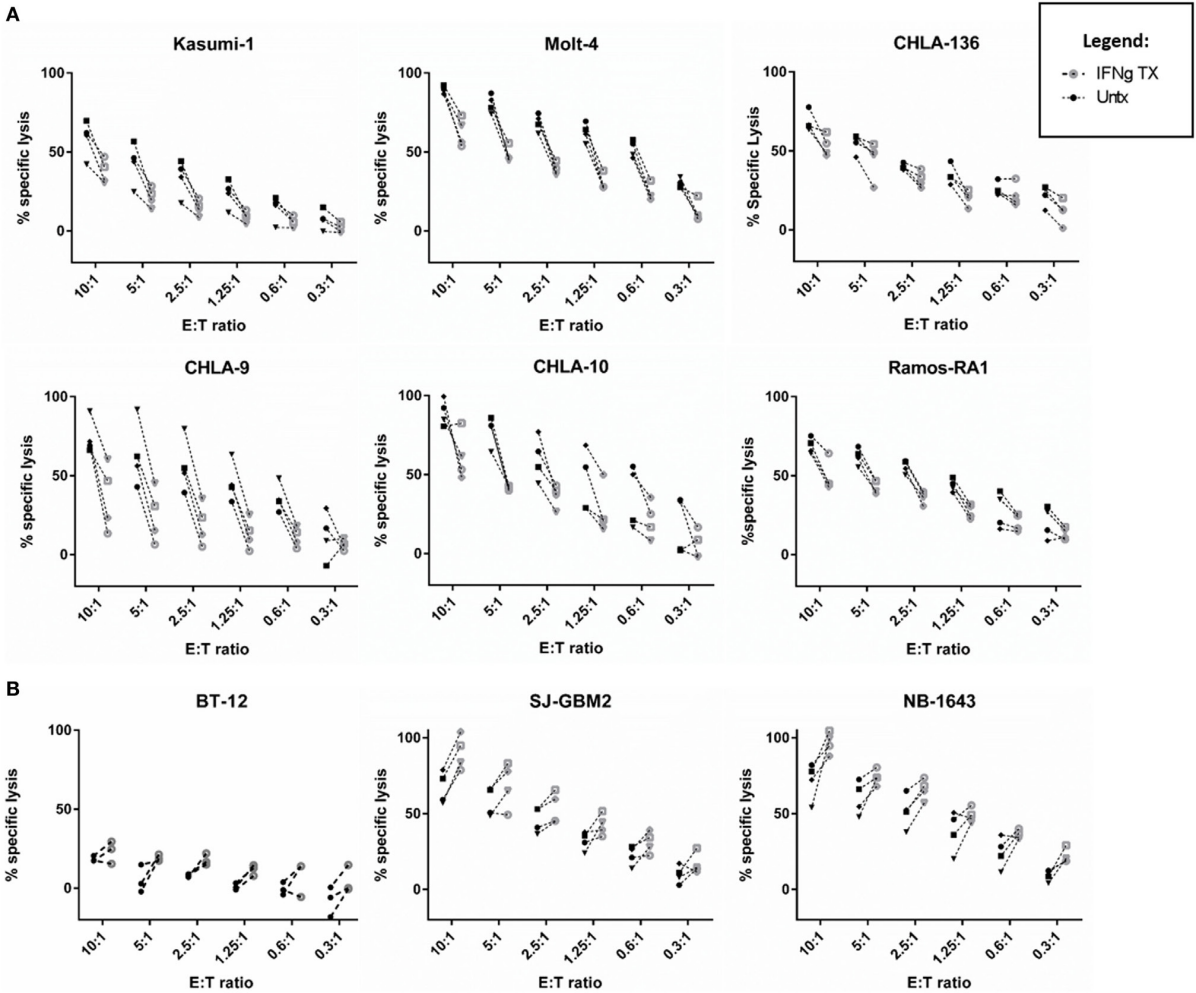
**IFNγ has a variable impact on natural killer (NK) cell-mediated cancer lysis**. NK cell cytotoxic activity toward IFNγ-treated (gray) and -untreated (black) cancer cells was evaluated at six different effector to target (E:T) ratios using calcein release assays. **(A)** Cell lines showing decreased lysis [leukemia (Molt-4, Kasumi-1), Ewing sarcoma (CHLA-9, CHLA-10), lymphoma (Ramos-RA1), and neuroblastoma (NB) (CHLA-136)] and **(B)** cell lines showing increased lysis [brain tumors (BT-12, SJ-GBM2) and NB (NB1643)] after IFNγ treatment. The four NK cell donors are represented for all cell lines, except for BT-12 with three donors represented.

We determined whether tumor IFNγ treatment affected NK cell-mediated lysis for 22 pediatric cancer cell lines from the PPTP *in vitro* panel. Tumor cell lines evaluated were derived from brain tumors, EWS, NB, leukemia, lymphoma, and RMS. The Rh18 cell line was excluded because of repeated problems maintaining the cell line in culture. We observed that 6 of the 22 cell lines evaluated show a significant decrease in NK cell-mediated lysis after IFNγ treatment (Figure 1). The group of cell lines with decreased lysis after IFNγ treatment includes leukemia cell lines Molt-4 (*p* = 0.0030) and Kasumi-1 (*p* = 0.0231), EWS cell lines CHLA-9 (*p* = 0.0037) and CHLA-10 (*p* = 0.0292), lymphoma cell line Ramos-RA1 (*p* = 0.0007), and NB cell line CHLA-136 (*p* = 0.0065). The NB cell line NB-EBc1 appears to have a decreased lysis after treatment, however, it was not statistically significant. When decreased lysis after treatment was observed, it was consistent for all four donors tested and across E:T ratios (Figure 2A).

By contrast, two of the cell lines showed an increase in NK cell-mediated lysis after IFNγ treatment. These cell lines include the glioblastoma cell line SJ-GBM2 (*p* = 0.0155) and the NB cell line NB1643 (*p* = 0.0371) (Figure 1). In addition, although the brain tumor cell line BT-12 shows a non-significant increase in sensitivity, we observed that for three of the four NK cell donors there was an increased sensitivity after IFNγ treatment, this increase is significant if these three donors are evaluated (*p* = 0.0061). Increased lysis after IFNγ treatment was a consistent finding with most donors mostly at high NK cell doses (Figure 2B). The remaining cell lines evaluated showed no differences in NK cell-mediated lysis after IFNγ treatment, and this group includes cell lines corresponding to all the tumor types evaluated (Figure 1). After stratifying our data by tumor type, we can observe that the only tumor type for which IFNγ had no effect on lysis was RMS. In EWS, leukemia, and lymphoma IFNγ had no effect or resulted in decreased lysis, whereas IFNγ had no effect or resulted in increased lysis for brain tumors. Interestingly, for NB cell lines, the effect of IFNγ on NK cell-mediated lysis was variable. Lysis of some NB cell lines was unaffected by IFNγ treatment, some became more resistant, and some more sensitive.

### IFNγ Alters Surface Expression of NK Cell Ligands in Pediatric Cancer Cell Lines

**Figure 3:**
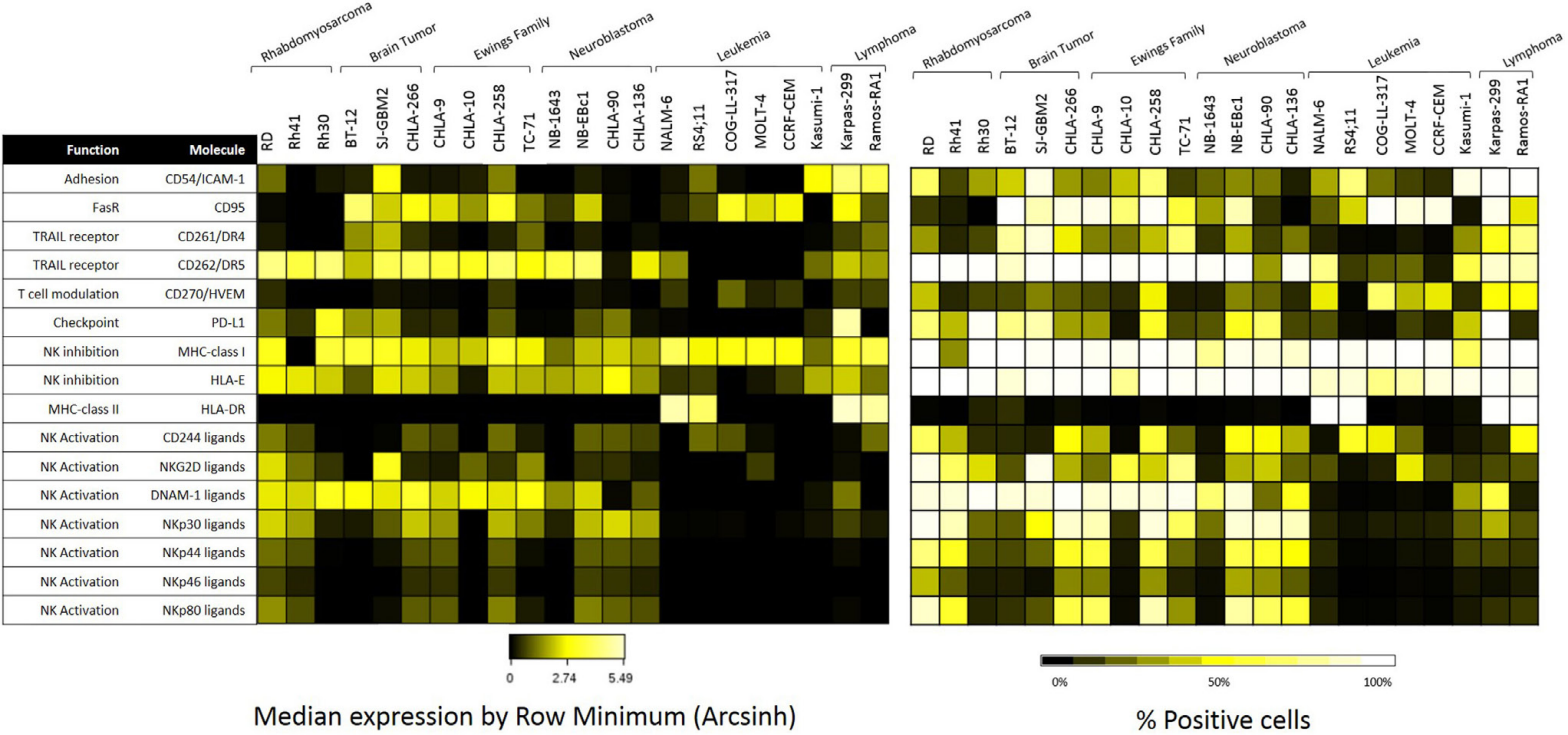
**Expression of natural killer (NK) cell ligands in pediatric cancer cell lines**. Pediatric cancer cell lines were evaluated by mass cytometry for the baseline expression level of multiple NK cell ligands. The evaluated markers and their function can be found at the table above. Left panel: median expression level normalized by row minimum (obtained by evaluating the arcsinh ratio of the median expression compared to the row minimum). Right panel: percent positive cells for each given marker.

The 22 pediatric cancer cell lines obtained from the PPTP *in vitro* panel were evaluated by mass cytometry for the expression of 16 NK cell ligands. Baseline expression of NK cell ligands in terms of median expression and percentage positive cells is provided in Figure 3. The inhibitory ligands MHC-class I and HLA-E are homogeneously expressed (>70%) for all PPTP cell lines with the exception of the RMS cell line Rh41 where MHC-class I was expressed in only 35% of the cells (Figure 3, right panel). Interestingly, we observe that solid tumor cell lines (RMS, brain tumor, EWS, and NB) have higher median expression levels of TRAIL receptor CD262/DR5, and ligands for the activating receptors NKG2D, DNAM-1, and NCRs, when compared to leukemia cell lines (Figure 3, left panel).

**Figure 4:**
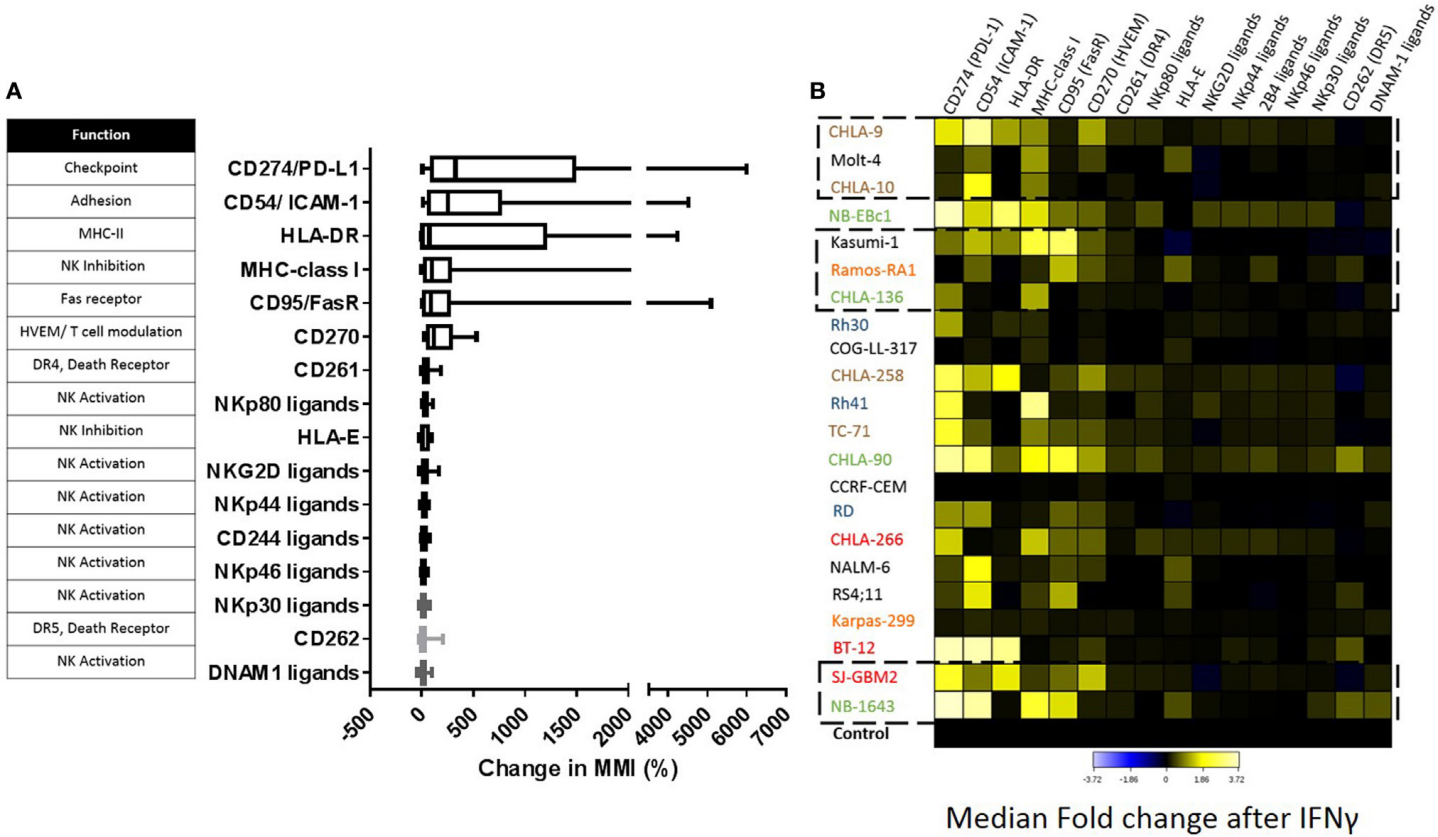
**Impact of IFNγ treatment on the surface expression of natural killer (NK) cell ligands for pediatric cancer cell lines**. **(A)** Change in expression level [mean mass intensity (MMI)] was determined by quantifying the mean of each parameter at baseline and after IFNγ treatment for each cell line. Percent change in MMI after IFNγ treatment was calculated for each cell line and box plots were generated for each parameter using data obtained from all 22 cell lines. **(B)** Fold change in median expression for each parameter in all cell lines. Heat corresponds to the fold change in median expression after IFNγ treatment (obtained as the arcsinh ratio of median expression for the given markers compared to untreated cell line control). Cancer type color coding is the same as in Figure 1. Squares with dashed lines indicate cancer cell lines with significant changes in NK cell-mediated lysis after IFNγ treatment.

**Figure 5:**
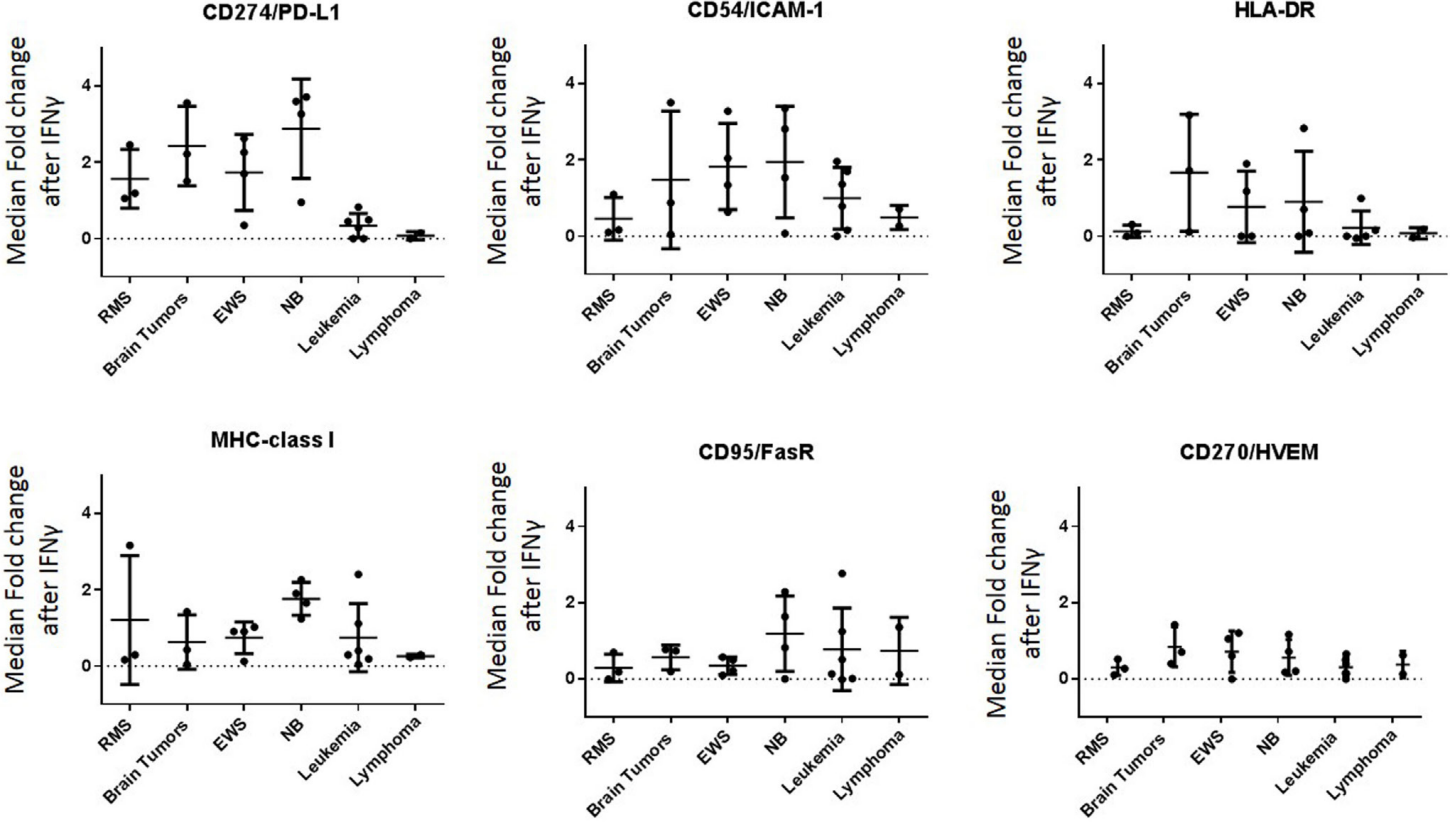
**Major ligands affected by IFNγ classified by tumor type**. IFNγ induced changes in NK cell ligand expression were evaluated by cancer type. *Y*-axis corresponds to the fold increase in marker median expression after IFNγ treatment, this was obtained by evaluating the arcsinh ratio of median expression for the given marker compared to untreated cell line.

Baseline expression levels of NK cell ligands were compared to the levels after IFNγ treatment and changes were quantified. The percentage change in mean mass intensity (MMI) after IFNγ treatment was determined for each of the 22 cell lines (Figure 4A). CD274/PD-L1, CD54/ICAM-1, HLA-DR, MHC-class I, CD95/FasR, and CD270/HVEM were the most affected by IFNγ (Figure 4A), with at least three cell lines showing fivefold increase or more in MMI. Data were also evaluated in terms of median expression. IFNγ induced changes in median expression were quantified, and a heatmap corresponding to the fold increase in median expression (obtained from the arcsinh ratio of median) was generated (Figure 4B). The changes in median expression were similar to changes in mean expression. CD274/PD-L1, CD54/ICAM-1, HLA-DR, MHC-class I, CD95/Fas, and CD270/HVEM were the markers mostly upregulated by IFNγ (Figure 4B). When stratified by tumor type (Figure 5; Figure S2 in Supplementary Material), we observe that PD-L1 was most upregulated by IFNγ in solid tumor cell lines (RMS, brain, EWS, and NB), but was relatively unaffected on leukemia and lymphoma cell lines. ICAM-1 upregulation was higher for brain tumors, EWS, NB and leukemia, and lower for RMS and lymphomas. HLA-DR was upregulated by IFNγ on some brain tumor, EWS, and NB cell lines. MHC-class I upregulation was variable even within the same tumor type. However, IFNγ-induced MHC-class I upregulation was consistently observed in all NB cell lines. Finally, CD270/HVEM upregulation was induced by IFNγ in some brain tumor, EWS, and NB cell lines. Our data show that lymphoma cell lines were the least affected by IFNγ in terms of NK cell ligand expression (Figure 5; Figure S2 in Supplementary Material).

### IFNγ-Induced Upregulation of MHC-Class I and ICAM-1 Correlates with Changes NK Cell-Mediated Lysis

**Figure 6:**
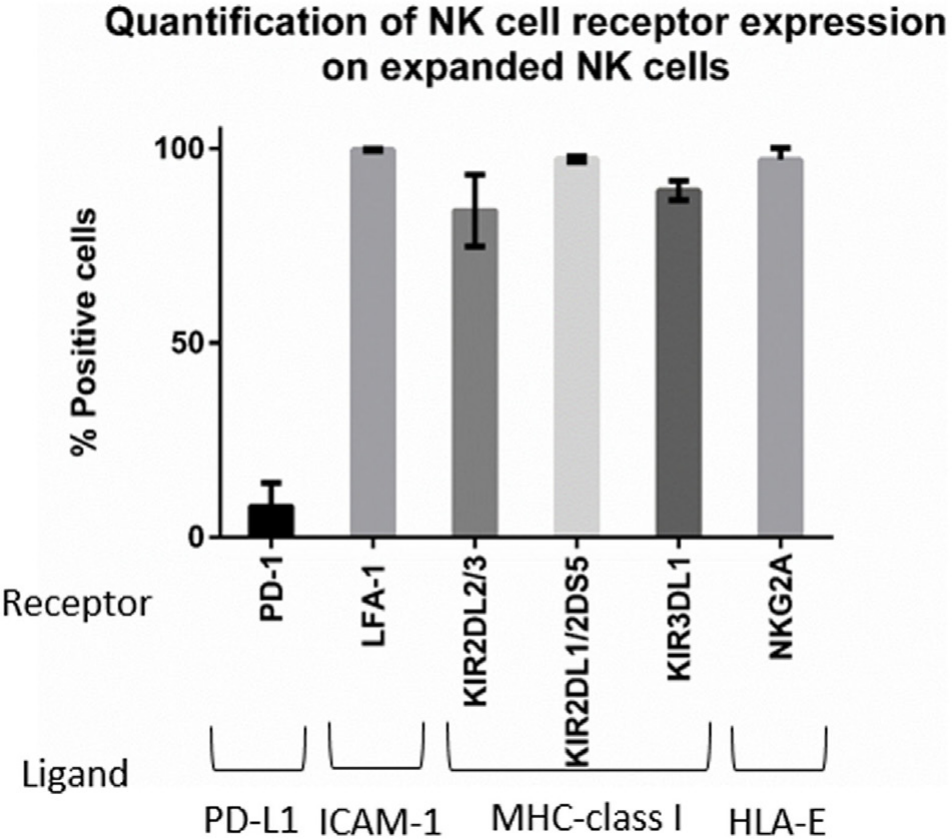
**Phenotyping of expanded natural killer (NK) cells**. Expression of PD-1, LFA-1, killer cell immunoglobulin-like receptors (KIRs), and NKG2A on the expanded NK cells was obtained by mass cytometry (*n* = 4 donors).

Next, we wanted to determine whether there was a correlation between changes in ligand expression after IFNγ treatment and changes in NK cell-mediated tumor lysis. We first assessed our expanded NK cells for expression of the receptors corresponding to the most upregulated ligands—PD-L1, ICAM-1, and MHC-class I. Expression of PD-1, the receptor for PD-L1, was observed on only 7% of the expanded NK cells (Figure 6). This low percentage of PD-1+ NK cells in our expanded product suggests that PD-L1 upregulation is unlikely to play a role in the IFNγ-induced changes in lysis we observed.

**Figure 7:**
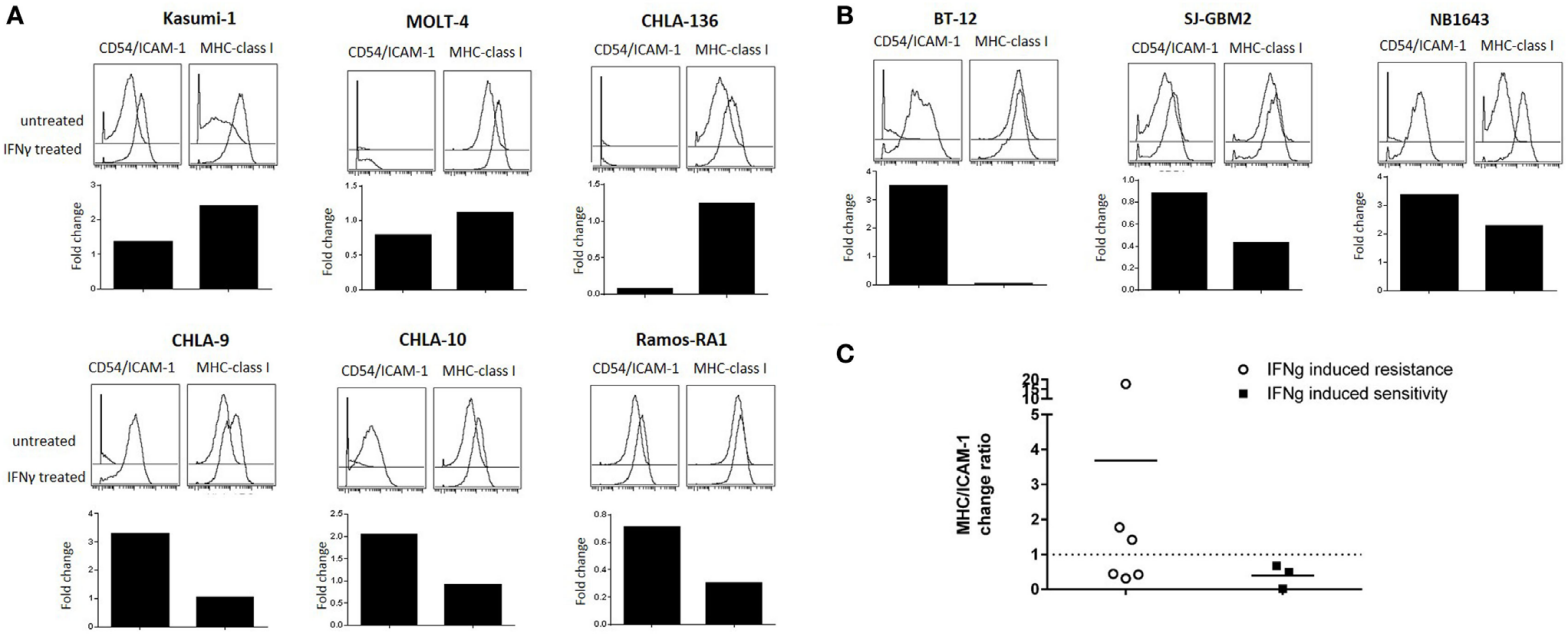
**Effect of IFNγ treatment on intercellular adhesion molecule 1 (ICAM-1) and major histocompatibility complex (MHC)-class I expression for cancer cell lines with altered sensitivity**. The effect of IFNγ on ICAM-1 and MHC-class I expression for cell lines in which IFNγ treatment conferred **(A)** resistance or **(B)** sensitivity to NK cell-mediated lysis. Histograms compare ICAM-1 (left) and MHC-class I (right) expression for untreated cancer cells (top) and IFNγ-treated cells (bottom). Bar graphs represent ICAM-1 and MHC-class I fold change in median expression after IFNγ treatment. **(C)** Ratio of MHC-class I/ICAM-1 change after IFNγ treatment for cell lines with altered sensitivity. Fold change in median expression after IFNγ treatment for each parameter was quantified and the ratio was calculated. Ratio <1 indicates higher ICAM-1 upregulation and ratio >1 indicates higher MHC-class I upregulation after IFNγ treatment.

By contrast, LFA-1, the integrin that binds ICAM-1, was expressed in 99.6% of our expanded NK cells (Figure 6), and inhibitory KIR receptors were also highly expressed in our expanded NK cells. KIR2DL2/3 expression was 83.9%, KIR2DL1/2DS5 was 97.18%, and KIR3DL1 was 89.15% (Figure 6). Also we observed expression of the inhibitory receptor NKG2A in 97% of expanded NK cells. Knowing that the majority of our expanded NK cells express LFA-1, KIR, and NKG2A receptors, we focused on changes in ICAM-1 and MHC-class I expression (Figure 7). The ratio of change in MHC-class I over change in ICAM-1 after IFNγ treatment was evaluated and plotted for cell lines with altered sensitivity (Figure 7C). We observed that all the cell lines that became more resistant after IFNγ treatment had an increase in MHC-class I expression (Figures 2A and 7A). However, MHC-class I upregulation was also observed in some of the cell lines with increased sensitivity after IFNγ treatment (Figures 2B and 7B). Interestingly, MHC-class I/ICAM-1 change ratio was <1 and, indicating that upregulation of ICAM-1 exceeded MHC-class I upregulation, for all the cell lines with increased sensitivity after IFNγ treatment (Figures 7B,C). The opposite pattern, with an MHC-class I/ICAM-1 change ratio >1 and MHC-class I upregulation exceeding ICAM-1 upregulation, was observed in three of the six cell lines where IFNγ induced resistance (Kasumi-1, MOLT-4, and CHLA-136) (Figures 7A,C). However, ICAM-1 upregulation exceeded MHC-class I upregulation (ratio <1) in the EWS cell lines CHLA-9 and CHLA-10 and the lymphoma cell line Ramos-RA1, for which IFNγ treatment resulted in decreased NK cell-mediated lysis.

### IFNγ-Induced ICAM-1 Upregulation Increases Conjugate Formation for Cell Lines with Increased Sensitivity

**Figure 8:**
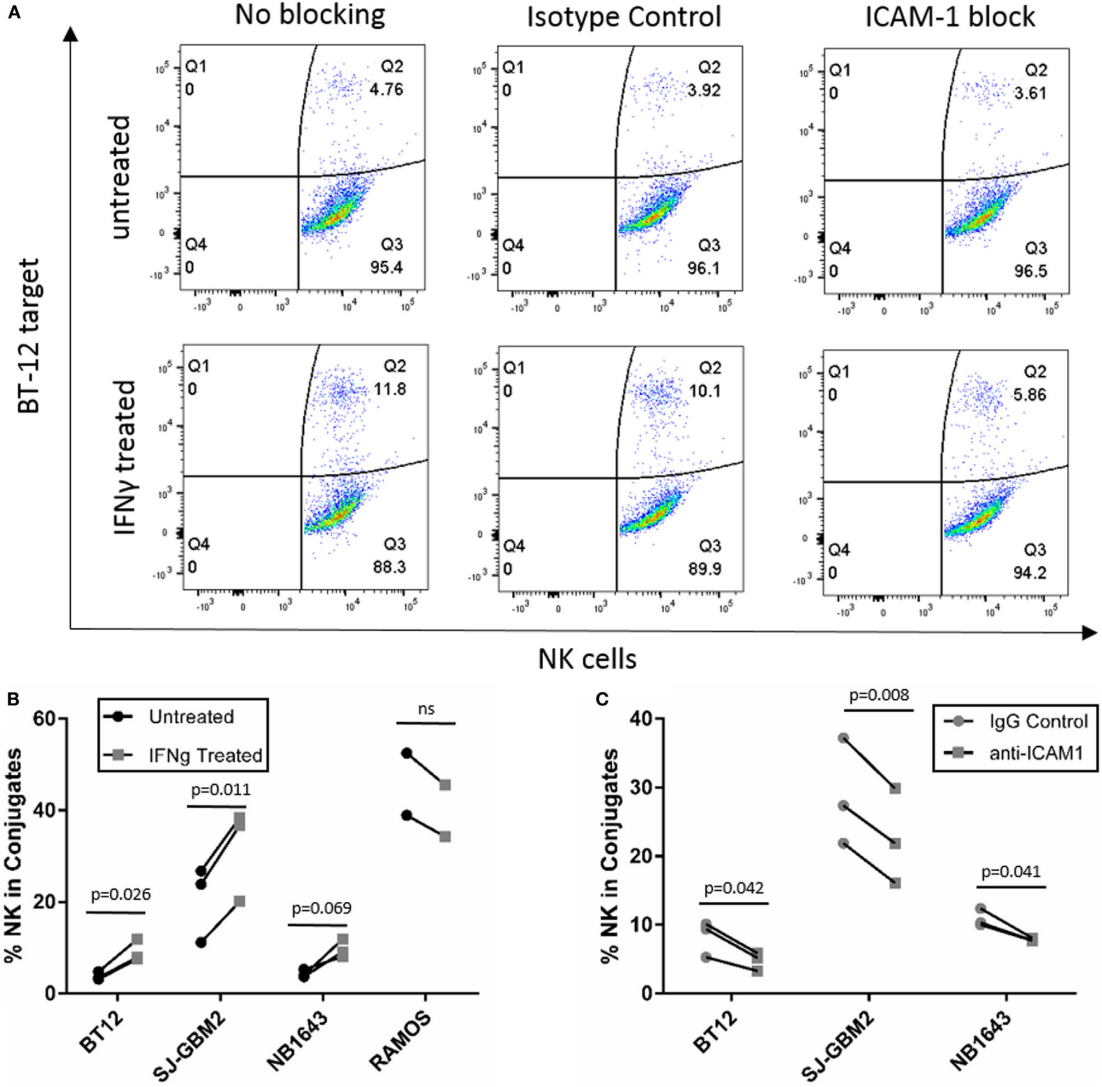
**Conjugation assay for cell lines with increased sensitivity after IFNγ treatment**. **(A)** *y*-Axis represents fluorescence of labeled target cells and *x*-axis represents fluorescence of labeled natural killer (NK) cells, and Q2 indicates dual fluorescence of NK cells conjugated with target cells. **(B)** Quantification of %NK cells in conjugate with BT-12, SJ-GBM2, NB1643, and Ramos-RA1. **(C)** Quantification of %NK cells in conjugate after ICAM-1 block for IFNγ-treated BT-12, SJ-GBM2, and NB1643 (*n* = 3 donors, Student’s *t-*test).

Since IFNγ is known to enhance MHC-class I expression, resulting in resistance of target cells to NK cell-mediated lysis (9, 10), we investigated possible mechanisms for the enhanced sensitivity observed after treatment for the cell lines SJ-GBM2, NB1643, and BT-12. These showed increased sensitivity to NK cell-mediated lysis after IFNγ treatment despite presence of high levels of MHC-class I. Since upregulation of the adhesion molecule ICAM-1 exceeded MHC-class I upregulation for these tumors, we determined whether IFNγ-mediated ICAM-1 upregulation was capable of overcoming MHC-class I inhibition through increased conjugate formation between the NK cells and the target cells (Figure 8A). For comparison, we also evaluated IFNγ-mediated changes in conjugate formation between NK cells and Ramos-RA1, a cell line having high ICAM-1 expression but for which IFNγ resulted in more resistance. We observed that treatment of BT-12, SJ-GBM2, and NB1643 with IFNγ resulted in increased conjugate formation (Figure 8B) and this was statistically significant for BT-12 (*p* = 0.026) and SJ-GBM2 (*p* = 0.011). By contrast, IFNγ treatment did not increase conjugate formation for Ramos-RA1. Next, to confirm whether the increased conjugate formation was specifically mediated by ICAM-1 upregulation, we used monoclonal antibodies to block ICAM-1 on IFNγ-treated cells (BT-12, SJ-GBM2, and NB1643). Blocking of ICAM-1 resulted in a significant decrease in conjugate formation for BT-12 (*p* = 0.042), SJ-GBM2 (*p* = 0.008), and NB1643 (*p* = 0.041) (compared to isotype control, Figure 8C).

## Discussion

Natural killer cells are an attractive approach for cancer immunotherapy. Our laboratory has developed an *ex vivo* NK cell expansion platform that allows us to generate large quantities of NK cells for patient infusion. We have shown that our expanded NK cells, currently used in several clinical trials for myeloid malignancies and posterior fossa tumors (NCT01787474, NCT01904136, NCT01823198, NCT02271711), secrete large amounts of IFNγ compared to primary NK cells (8). Similarly, memory-like NK cells used for adoptive transfer to AML patients exhibited enhanced IFNγ production when compared to control NK cells (18). Due to the high levels of IFNγ secreted by NK cell infusion products, we sought to determine the effects of IFNγ on NK cell interactions with the tumor cells.

Through this study we evaluated a broad selection of pediatric tumor cells, representing at least six different types of malignancies, for the effect of IFNγ on their sensitivity to NK cell-mediated lysis using expanded NK cells from four independent donors. This enabled a broad study across tumor types demonstrating opposing effects of IFNγ, a finding that may not have been evident in other studies that focus on a single tumor type or modulation of a single surface ligand. Of the 22 cell lines evaluated, six showed a significant decrease in NK cell-mediated lysis after IFNγ treatment, including leukemia, EWS, lymphoma, and NB cells. By contrast, treatment with IFNγ resulted in enhanced sensitivity to NK cells for three cell lines, BT-12, SJ-GBM2, and NB1643 (two brain tumors and a NB, respectively). For the remaining cell lines, IFNγ treatment did not significantly affect NK cell-mediated lysis, though some showed trends that may be significant with more donor replicates. The effect of IFNγ treatment was variable within the same tumor type, with the exception of RMS cell lines for which IFNγ treatment had no effect. These results suggest that the effect of IFNγ on NK cell-mediated lysis of tumor cells is variable and cell line dependent. Our findings warrant more focused investigation and validation for specific tumor types using primary tumor samples or patient-derived xenografts where feasible.

To better understand this variability, we used mass cytometry to evaluate the effect of IFNγ on expression of NK cell ligands by cancer cells. First, we observed broad heterogeneity in baseline expression levels of ligands between and within tumor types (Figure 3B). Solid tumor cell lines had higher median expression of TRAIL receptor CD262/DR5 and ligands for the activating receptors NKG2D, DNAM-1, and NCRs, compared to leukemia cell lines (Figure 3A). This suggests NK cells may be a promising therapy for solid tumors. We also observed that none of these ligands were downregulated by IFNγ; however, CD274/PD-L1, CD54/ICAM-1, HLA-DR, MHC-class I, CD95/FasR, and CD270/HVEM were upregulated in a variety of tumor types. Other than CD270/HVEM, these findings are consistent with previously published studies (11, 12, 19–24). HVEM is involved in T cell regulation (25), but no studies have yet reported regulation of this ligand by IFNγ and its role in NK cell biology has not been well described.

Although IFNγ-mediated upregulation of MHC-class I, PD-L1, and ICAM-1 has been previously described, this study uncovers the variability of IFNγ responses across different pediatric tumor types. In terms of PD-L1, our results show upregulation by IFNγ for most pediatric solid cancers (RMS, brain tumors, EWS, and NB), but no effect on pediatric leukemia and lymphoma cells. On the other hand, the adhesion molecule ICAM-1 was upregulated by IFNγ on brain tumors, EWS, NB, and leukemia, but not on RMS and lymphoma cells. Although IFNγ-induced ICAM-1 upregulation has been previously described in NB and leukemia cells (11, 12), to our knowledge no studies have shown its upregulation on EWS.

According to our data, the ligands most upregulated by IFNγ were PD-L1, ICAM-1, and MHC-class I; therefore, we determined whether changes in their expression correlated to changes in tumor lysis after IFNγ treatment. PD-1/PD-L1 is associated with immune cell suppression; however, we observed that <7% of expanded NK cells express PD-1. In addition, changes in expression of PD-L1 did not correlate with changes in cytotoxicity, suggesting that PD-L1 did not play a role in our model. By contrast, changes in ICAM-1 and MHC-class I cooperate in affecting NK cell responsiveness. For the cell lines in which IFNγ increased sensitivity to NK cells, we observed ICAM-1 upregulation exceeding MHC-class I upregulation (MHC-class I/ICAM-1 < 1). This suggests that the LFA-1/ICAM-1 interaction augments NK cell-mediated lysis even in the presence of high levels of inhibitory MHC-class I molecules. MHC-class I molecules mediate NK cell inhibition through binding with KIRs (HLA-A, HLA-B, HLA-C) and NKG2A (HLA-E). The anti-MHC-class I antibody clone used in this study, W6/32, recognizes both classical (HLA-A, HLA-B, HLA-C) and non-classical (HLA-E) HLA (26). We observed that for three of the six cell lines with increased resistance after IFNγ treatment, MHC-class I upregulation exceeded ICAM-1 upregulation (MHC-class I/ICAM-1 >1). This suggests that the NK cell balance was shifted toward inhibition due to the increased expression of MHC-class I, which binds NK cell inhibitory receptors. However, in the other three cell lines with increased resistance after IFNγ treatment we observed that ICAM-1 upregulation exceeded MHC-class I upregulation (MHC-class I/ICAM-1 <1) for which this model would have predicted increased sensitivity after IFNγ treatment. Our results suggest that the differential effects on MHC-class I and ICAM-1 expression can explain some but not all of the effects of IFNγ on sensitivity to NK cell lysis.

We investigated possible mechanisms for the enhanced sensitivity observed after IFNγ treatment for the cell lines SJ-GBM2, NB1643, and also BT-12. After IFNγ treatment these cell lines showed increased sensitivity to NK cell-mediated lysis, even in the presence of high levels of MHC-class I (Figures 2B and 7B). Since IFNγ treatment caused ICAM-1 upregulation exceeding MHC-class I upregulation in these cells lines (MHC-class I/ICAM-1 <1), we evaluated conjugate formation after IFNγ treatment. Our results show an increase in the formation of conjugates between the NK cells and the target cells SJ-GBM2, BT-12, and NB1643 after IFNγ treatment, and conjugate formation was decreased for IFNγ-treated cells after blocking ICAM-1 on the target cells. Ramos-RA1 did not show increased conjugate formation or killing in response to IFNγ, possibly because it has such high baseline ICAM-1 expression and conjugate formation. This suggests that the mechanism of increased sensitivity from IFNγ treatment is, at least in part, mediated by increased ICAM-1 upregulation leading to enhanced effector-target conjugation.

Our study uncovers the complexity behind cancer cell responses to IFNγ. Published literature has shown increased resistance to NK cell-mediated lysis due to MHC-class I upregulation in some cancers (9, 10), but increased sensitivity to NK cell-mediated lysis due to ICAM-1 upregulation in others (11, 12). These studies reported IFNγ effects on MHC-class I or ICAM-1 expression individually, and the contradictory results stem from analysis of a small number of different targets or focus on particular tumor types. This is, to our knowledge, the first study to evaluate the effect of IFNγ on a broad range of NK cell ligands and across nearly two dozen cell lines representing six broad tumor types. We observed variable effects of IFNγ on tumor sensitivity to NK cells that are cell line dependent and attributable to individual MHC-class I and ICAM-1 responses, suggesting that IFNγ effects on cancer cell sensitivity to NK cells should not be broadly generalized, although further experiments with cell lines and patient samples may determine whether specific tumor types have generalizable IFNγ responses. Our data suggest that increased IFNγ secretion from expanded NK cells can mediate ICAM-1 upregulation and enhanced NK cell conjugate formation in brain tumors and NB, enhancing NK cell activity in adoptive immunotherapy. Thus, a better understanding of the effects of NK cell-mediated inflammation on the tumor microenvironment is essential to optimize cellular immunotherapy of cancer.

## Author Contributions

Conceived and designed the experiments: AA-L, VS, EK, and DL. Performed the experiments and acquired data: AA-L, VS, and ZV. Analyzed the data: AA-L, EK, and DL. Wrote the paper: AA-L and DL. All authors read and accepted the final version of the manuscript.

## Conflict of Interest Statement

DL declares a potential conflict of interest in licensing of intellectual property to Intrexon Corporation and Ziopharm Oncology, and equity/leadership in Cyto-Sen Therapeutics. The other authors declare no conflict of interest.
