# Supplementary material for: Interferon Gamma Induces Changes in Natural Killer (NK) Cell Ligand Expression and Alters NK Cell-Mediated Lysis of Pediatric Cancer Cell Lines
Source: Front Immunol. 2017 Apr 6;8:391. doi: 10.3389/fimmu.2017.00391 (PMC5382194; doi:10.3389/fimmu.2017.00391)
Supplement: Supplementary file 1 [file Table_1.PDF]

Supplementary Material

IFNγ Induces Changes in NK Cell Ligand Expression and Alters NK Cell Mediated Lysis of Pediatric Cancer Cell Lines

Arianexys Aquino-López[1], Vladimir V. Senyukov, Zlatko Vlasic, Eugenie S. Kleinerman and Dean A. Lee

Correspondence: dean.lee@nationwidechildrens.org

	Isotope	Antibody or Chimeric protein (Fc)	Antibody Clone	Source	Catalog #
Panel for tumor cells	149Sm	CD244/2B4_Fc	-	R&D	1039-2B-050
	150Nd	HLA-E	3D12	BioLegend	342602
	152Sm	NKp80_Fc	-	R&D	1900-NK-050
	154Sm	NKp30_Fc	-	SinoBiological	10480-H03H
	156Gd	NKp44_Fc	-	R&D	2249-NK-050
	161Dy	CD261/DR4	DJR1	BioLegend	307202
	162Dy	CD54/ICAM-1	HCD54	BioLegend	322702
	163Dy	CD262/DR5	DJR2-4(7-8)	BioLegend	307402
	165Ho	DNAM-1_Fc	-	R&D	666-DN-050
	166Er	MHC-class I	W6/32	BioLegend	311402
	168Er	NKG2D/CD314_Fc	-	R&D	1299-NK-050
	169Tm	NKp46_Fc	-	R&D	1850-NK-025
	170Er	CD270	122	BioLegend	318802
	172Yb	CD274/PD-L1	29E.2A3	BioLegend	329702
	174Yb	HLA-DR	L243	BioLegend	307602
	175Lu	CD95/Fas	DX2	BioLegend	305602
	**Isotope**	**Antibody**	**Antibody Clone**	**Source**	**Catalog #**
Panel for NK cells		KIR3DL1-PE	DX9	Miltenyl Biotec	130-092-473
	141Pr	Anti-PE	PE001	BioLegend	408102
		KIR2DL1-FITC	143211	R&D	FAB1844F
	144Nd	Anti-FITC	FIT-22	DVS-Fluidigm	3144006B
	151Eu	CD3	UCHT1	BioLegend	300443
	162Dy	CD56	NCAM16.2	BD	559043
	166Er	CD159a (NKG2A)	131411	R&D	MAB1059
	168Er	CD11a (LFA-1)	HI111	BioLegend	301202
		KIR2DL2/3-APC	DX27	Miltenyl Biotec	130-092-617
	170Er	Anti-APC	APC003	BioLegend	408002
	175Lu	CD279 (PD-1)	J105	MBL	D133-3

Table S1. CyTOF Panels for cancer cell and NK cell staining. The staining panel for cancer cells includes antibodies against NK cell ligands when known and existing as a single ligand for an NK cell receptor, or chimeric receptor:IgG-Fc fusion proteins when ligands are unknown or if multiple ligands exist for a receptor. Antibodies and fusion proteins were conjugated with heavy metals and used for identification of ligands on cancer cells. The staining panel for NK cells includes metal-conjugated antibodies or fluorophore-conjugated antibodies with the corresponding secondary metal-conjugated anti-fluorophore antibody.
