# Supplementary material for: Interferon Gamma Induces Changes in Natural Killer (NK) Cell Ligand Expression and Alters NK Cell-Mediated Lysis of Pediatric Cancer Cell Lines
Source: Front Immunol. 2017 Apr 6;8:391. doi: 10.3389/fimmu.2017.00391 (PMC5382194; doi:10.3389/fimmu.2017.00391)
Supplement: Supplementary file 2 [file Image_1.PDF]

Supplementary Material

IFNγ Induces Changes in NK Cell Ligand Expression and Alters NK Cell Mediated Lysis of Pediatric Cancer Cell Lines

Arianexys Aquino-López[1], Vladimir V. Senyukov, Zlatko Vlasic, Eugenie S. Kleinerman and Dean A. Lee

Correspondence: dean.lee@nationwidechildrens.org

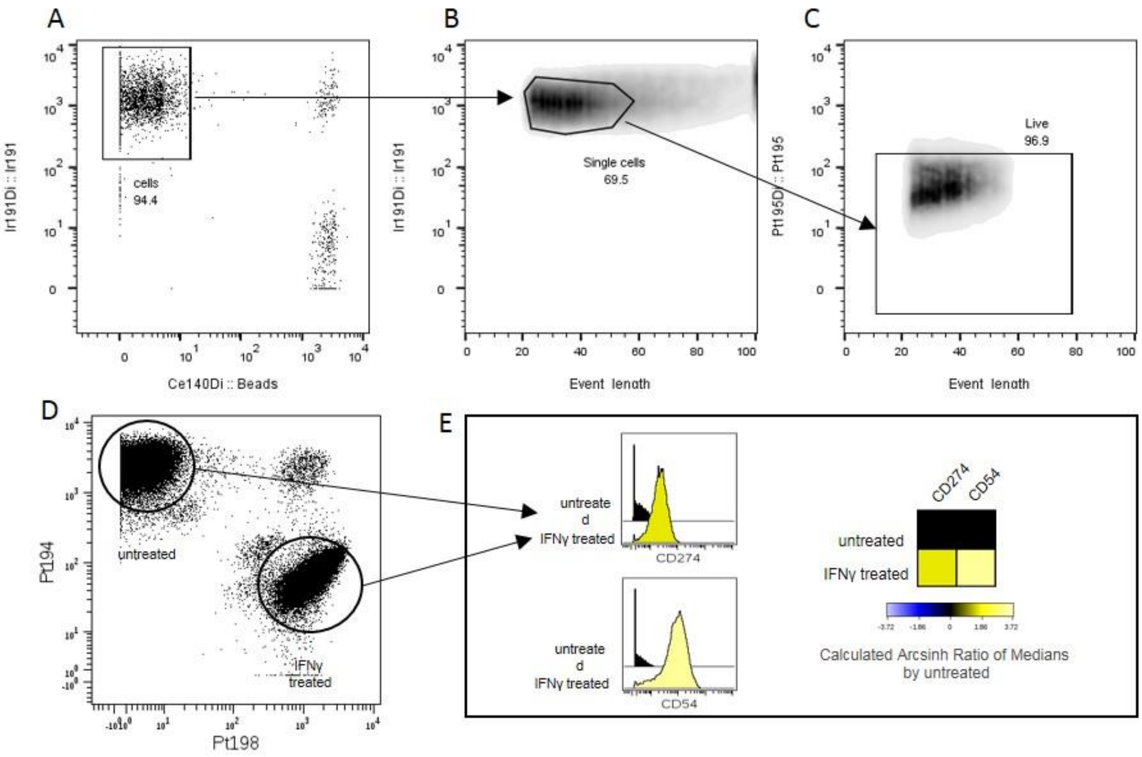

Figure S1. Gating strategy and de-barcoding of untreated and IFNγ treated cancer cells. (A) Cells were gated to remove beads from the anlaysis followed by (B) gating for single cells and removal of aggregates**. (C)** Dead cells exclusion. Live, single cells were exported as FCS files into Cytobank for de-barcoding **(D)** of treatment conditions. Untreated cells were positive for Pt-194 and IFNγ treated cells were positive for Pt-198. **(E)** Gated populations were analyzed and used for the generation of heat maps based on median expression of surface ligands.
