# Supplementary material for: Interferon Gamma Induces Changes in Natural Killer (NK) Cell Ligand Expression and Alters NK Cell-Mediated Lysis of Pediatric Cancer Cell Lines
Source: Front Immunol. 2017 Apr 6;8:391. doi: 10.3389/fimmu.2017.00391 (PMC5382194; doi:10.3389/fimmu.2017.00391)
Supplement: Supplementary file 3 [file Image_2.PDF]

Supplementary Material

IFNγ Induces Changes in NK Cell Ligand Expression and Alters NK Cell Mediated Lysis of Pediatric Cancer Cell Lines

Arianexys Aquino-López[1], Vladimir V. Senyukov, Zlatko Vlasic, Eugenie S. Kleinerman and Dean A. Lee

Correspondence: dean.lee@nationwidechildrens.org

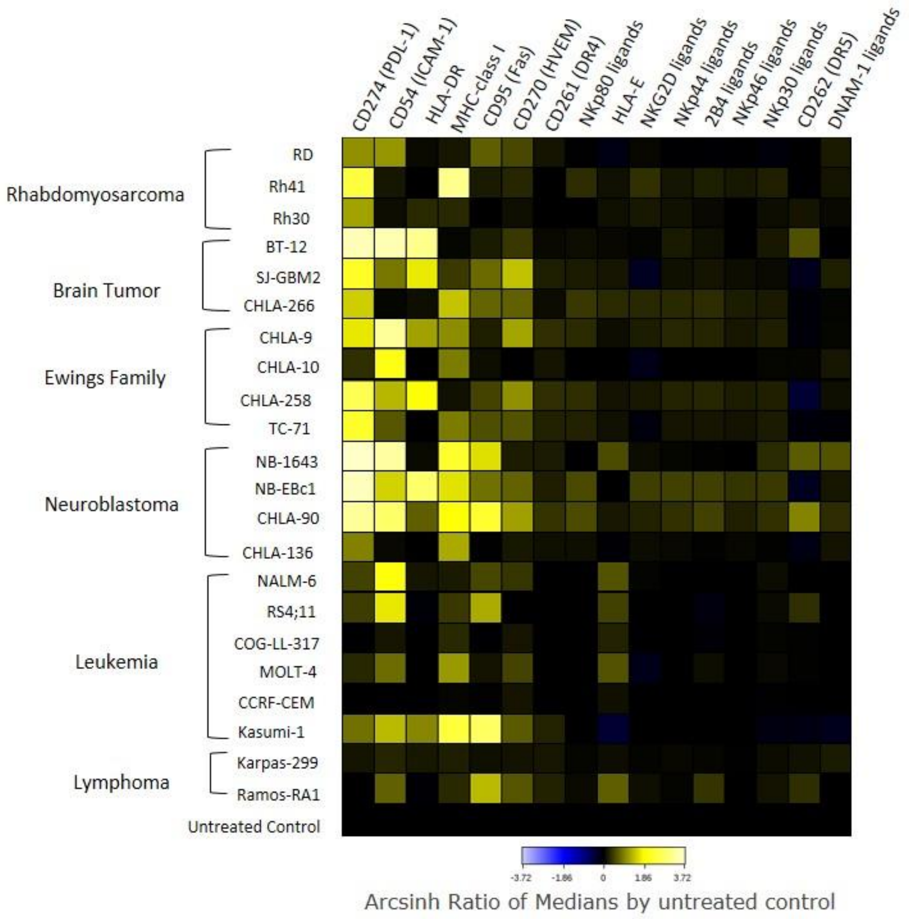

Figure S2. Impact of IFNγ treatment on the surface expression of NK cell ligands for pediatric cancer cell lines grouped by cancer type. IFNγ induced changes in NK cell ligand expression were evaluated in terms of median expression. Heat corresponds to the arcsinh ratio of median expression for the given markers compared to untreated cell line (Control).
